# Nitrogen Interactions with Phosphorus and Potassium for Optimum Crop Yield, Nitrogen Use Effectiveness, and Environmental Stewardship

**DOI:** 10.1100/tsw.2001.97

**Published:** 2001-10-11

**Authors:** Noble R. Usherwood, William I. Segars

**Affiliations:** Potash & Phosphate Institute, Norcross, GA, USA

**Keywords:** nitrogen, phosphorus, potassium, N-use effectiveness, nutrient interactions, environmental stewardship

## Abstract

The development of best management practices (BMPs) for optimum nitrogen (N) use by crops contributes to farm profitability, increased food and fiber production, and best stewardship of the environment and its resources. Such BMPs are both site- and crop-specific. Optimum N use by plants is influenced not only by climate and certain soil characteristics, but also by management practices such as tillage, time and method of N application, or positive interactions with nutrients and supporting cropping practices. Phosphorus (P) and potassium (K) are two of the nutrients essential for effective use of N by plants. Nitrogen interactions with P and/or K help to improve root system development, dry matter production, and other plant functions regulating crop yield and quality.

## INTRODUCTION

The importance of nitrogen (N) for the production of food, feed, and fiber throughout the world is well documented. Equally important is the fact that world need for food continues to increase as the amount of arable farmland continues to decline[1]. Socolow[2] suggests five principles for guiding the management of human impact upon the N cycle. One involves an emphasis on improving the efficiency of both food producers and consumers throughout the system. Less than half of the fixed N added by agriculture ends up in harvested crops, and less than one-quarter ends up in the food consumed. These scenarios present a meaningful challenge for farmers to produce more per unit of land area and to do it profitably by using environmentally sound agronomic practices. Such practices will be site-specific to the land and to the crop, and must originate from research and fit modern farming systems.

Environmental concerns relating N to air and water quality and the relationships of N to the sequestering of carbon in the soil are well documented[2,3]. Many sources of N are involved with such issues. One is reported to be in the form of commercial fertilizer N for the production of food to feed people and animals. Others sources of N include the natural decomposition of organic plant residues, the release of atmospheric N during electrical storms, organic N discharge from billions of wild animals and birds, and the application of animal manures and municipal waste treatment products to farm fields.

## DISCUSSION

Decades of field research on N from commercial fertilizer sources have generated a data base of knowledge capable of establishing site- and crop-specific best management practices (BMPs) for N use that are efficient, effective and most protective of the environment. Such BMPs must address differences among crops with respect to total need for N, as well as the need during specific stages of plant growth. They embrace soil analysis, consider N

release from crop residues and soil organic matter, and take into account soil physical and chemical characteristics that influence N movement in the soil, and they embrace the positive interactions of N with other nutrients such as phosphorus (P), potassium (K) and/or sulfur (S). By understanding interactions among these nutrients and other essential inputs, N-use effectiveness can be optimized as suggested by Socolow[2] with the result of improved crop productivity and improved environmental stewardship of land and resources.

Although N is the most recognized plant nutrient, it does not work alone in plant nutrition. The visual effect of N is quite noticeable because it imparts dark green color and rapid growth in a plant, or when N is in short supply, the plant becomes pale green in color and stunted in growth. A shortage of P, K, or S, however, can drastically alter the effectiveness of N applications. A shortage of S, for example, will often express plant symptoms similar to a shortage of N even though adequate amounts of N are present. One of the reasons is that both N and S are components of two essential amino acids, methionine and cysteine, the building blocks for plant protein. A shortage of S creates inefficient and ineffective use of N. In a similar manner, insufficient P and/or K will partially inhibit major N functions in plant growth and restrict N use efficiency and productivity by the plant.

Long-term research by University of Maryland scientist Dr. Allan Bandel illustrates the influence of P and K on grain production by corn receiving 160 lb of N per acre per year[4]. Table 1 shows the results of a 10-year study comparing N with and without P plus K in the nutrient-management program. When corn received only N, grain yields progressively declined as soil P and K reserves were utilized. Grain production over the 10-year period declined by 52% as a result of inadequate P and K.

In 1999, Ohio State University scientist Dr. Jay Johnson reported the influence of P and K on the grain yield of corn fertilized with a uniform rate of N[5]. As shown in Table 2, this long-term study illustrates the value of balanced plant nutrition during years of soil moisture stress. Grain yields actually declined when P was applied to K-deficient soils receiving adequate N. As soil test-K level increased, grain yield response to P increased dramatically. Balancing the applied N with P and K resulted in higher grain yields, and a greater amount of the applied N was absorbed by the plant for residue and grain formation.

Florida researcher Dr. Fred Rhoads[6] evaluated the interaction of P and K with N by growing field corn on a sandy loam soil (testing low P, medium K) under center pivot irrigation. His findings also demonstrate the agronomic value of a balanced NPK fertilization program for corn (Table 3). Nitrogen-fertilized corn without supplemental P and K yielded 113 bu/acre. The addition of K without P increased corn yield nearly 23 bu/acre. The addition of P without K increased grain yield by 26 bu. When both P and K were applied, grain yield exceeded the combined individual yield responses from P and K by 15 bu/acre. By providing crop requirements for both P and K, the yield of grain per pound of N applied increased from 32 to 50 lb, an increase in N use effectiveness of more than 50%.

In a similar study in Illinois, the application of 120 lb/acre of potash to a soil testing high in K increased corn grain yield from 149 to 171 bu/acre. In this study, potash increased the pounds of grain produced per pound of N applied from 28 to 32 lb for nearly a 15% improvement.

As shown in Table 4, the optimum yield response from N applied to dryland wheat resulted when soil P was corrected as a yield limiting factor[7]. The quantity of wheat produced from

**TABLE 1**
**10-year Results of NPK vs. N Fertilization of Dryland Corn[4]**

| $N\text{-}P_2O_5\text{-}K_2O$ (lb/A/year) | Yield: lb grain/A for year 1 | 4 | 7 | 10 | 10 year total (*) |
|---|---|---|---|---|---|
| 160-160-160 | 8848 | 8600 | 6860 | 7,020 | 85600 (53.5) |
| 160-0-0 | 8200 | 4500 | 740 | 1180 | 41060 (25.7) |

*Note:* Pounds of grain per pound of N applied over the 10-year period.

**TABLE 2**
**Corn Response to Different Soil Test Levels in a Corn-Soybean Rotation[5]**

| P Soil Test (lb/A) | K Soil Test (lb/A): 178 | 209 | 298 |
|---|---|---|---|
| | Corn grain yield (bu/A) | | |
| 28 | 54 | 88 | 108 |
| 39 | 44 | 115 | 102 |
| 49 | 37 | 122 | 122 |

*Note:* Soil tests were taken after the 1998 soybean harvest (Ohio).

**TABLE 3**
**The Influence of P and K on Corn Response to Applied N[6]**

| Fertilizer treatment | Grain Yield (bu/A) | lb/A | lb grain/lb N |
|---|---|---|---|
| N only | 113 | 6328 | 32 |
| N + K | 136 (23 bu response to K) | 7616 | 38 |
| N + P | 139 (26 bu response to P) | 7784 | 39 |
| N + P + K | 177 (64 bu response to P & K) | 9912 | 50 |

**TABLE 4**
**The Effect of P on Dryland Wheat Response to Applied N[7]**

| Applied N (lb/A) | Applied $P_2O_5$ (lb/A) | Wheat Grain Yield | | Wheat Grain/N Ratio lb grain/lb N applied |
|---|---|---|---|---|
| | | bu/A | lb/grain/A | |
| 0 | 0 | 32 | 1920 | |
| 60 | 0 | 38 | 2280 | 38 |
| 60 | 60 | 58 | 3480 | 58 |

60 lb of applied N increased from 38 to 58 lb — a 52% increase — as a result of the application of P.

Nitrogen-use effectiveness can also be improved for forage crops. As shown in Table 5, Georgia researchers evaluated the importance of K to N-use by coastal bermudagrass[8]. The most economic rate of N for optimum yield and quality was determined to be 400 lb/acre when P and K were not limiting plant growth. When crop need for K was provided, forage yield increased from 5.2 to 7.5 t/acre, and total N uptake by the harvested forage increased dramatically. When K was inadequate, the forage absorbed only 75% of the N applied. With adequate K nutrition, bermudagrass roots captured 92% of the 400 lb/acre of applied N.

The benefits of balanced fertility and highly fertile soils are not restricted to yield improvement alone[9]. This was documented by the bermudagrass research conducted by Georgia scientists (Table 6). Bermudagrass grown on fertile soils had a deep and well-developed root system. More roots growing deeper into the soil enable plants to better utilize nutrients such as N and water positioned deep in the soil profile. More roots also mean more organic matter left in the soil. Research by the U.S. Department of Agriculture Agricultural Research Service's National Soil

**TABLE 5**
**Influence of K on N-Use Effectiveness of Coastal Bermudagrass[8]**

| $N-P_2O_5-K_2O$ (lb/A) | Forage Yield (tons/A) | Nitrogen uptake (lb/A) | Use Effectiveness (%) |
|---|---|---|---|
| 400-200-50 | 5.2 | 298 | 75 |
| 400-200-200 | 7.5 | 369 | 92 |

**TABLE 6**
**The Influence of Soil Fertility on Rooting Depth of Coastal Bermudagrass[9]**

| Soil Depth (inches) | Bermudagrass Roots (lb/A) | |
|---|---|---|
| | Low-fertility Soil | High-fertility Soil |
| 0 – 6 | 1160 | 2620 |
| 6 – 12 | 350 | 890 |
| 12 – 18 | 290 | 770 |
| 18 – 24 | 260 | 640 |
| 24 – 36 | 1280 | 1940 |
| **Total** | **3340** | **6860** |

Tilth Lab indicates that 75% of the new carbon (organic matter) comes from the roots and 25% from the crop residues[10]. By fertilizing soils low in P with adequate amounts of P, an improvement in soil organic matter from both roots and shoots will enhance soil quality. As crop residue is decomposed by soil microorganisms, soil particles are bound together into stable aggregates. This process improves soil structure, allows surface water to enter the soil with greater ease, and thereby decreases erosion.

Crops experiencing N, P, and/or K nutrition disorders might not always show visible distress signals. Many signals are related to nutrient function in plant growth. A shortage of a nutrient can decrease plant capacity to develop. Where two or more nutrients are involved with the same function, a shortage of one can initiate a reduction in effectiveness of the other even though it is present in sufficient amount; thus, balanced nutrition becomes fundamental to effective crop use of available N.

According to Smil[11], human activity has nearly doubled the amount of N entering the N biospheric cycle, and crop production being one of the large contributors. Of all the N inputs, about half are believed to be assimilated by harvested crops. The remainder become subject to loss to the atmosphere by volatilization and denitrification and to surface and ground waters through loss by leaching and erosion. In spite of the losses, the world's agricultural lands are presently N accumulators and the growing world population will require further increases in N use on croplands.

Science responds to this challenge through the identification of BMPs capable of improving crop use effectiveness and efficiency of applied and residual sources of N. A few of these, according to Munson and Runge[12] involve soil analysis for nutrients that interact with N; selection of N sources less subject to volatilization and loss by leaching; establishment of proper nutrient ratios; and better timing of nutrient applications in relation to crop need. In addition, the use of special ingredients added to N fertilizers, such as nitrification or urease inhibitors, is sometimes helpful in improving N availability to crops.

## CONCLUSION

As precision agricultural technology evolves, a greater amount of land area will likely utilize variable rate technology for the application of N and other crop production inputs. Adjustments in the genetic code of plants will likely develop crops with improved root systems, greater resistance and/or tolerance to stress, and other attributes related to improved N-use efficiency.

All of these new technologies and solutions are not without cost or without a time requirement for understanding, acceptance, and adoption by farmers of all nations. Also, the basic physiology set down for plant growth will not likely be altered appreciably. Each plant will continue to require each of the 17 essential elements for plant growth. Each plant will continue to require that these elements be available in the right quantities, at the right time and in the right form for the development of its full genetic potential for growth. For optimum use effectiveness of N in crop production, plants will continue to require a balance of nutrients, such as P and K.

## References

1. Usherwood, N.R., Dibb, D.W., and Darst, B.C. (2000) Fertilizers, food production and the environment. Proc. Am. Chem. Soc. Nat. Convention. Washington, D.C.
2. Socolow, R. H. (1999) Nitrogen management and the future of food: lessons from the management of energy and carbon. *Proc. Natl. Acad. Sci U.S.A.* **96,** 6001–6008.
3. Segars, W.I. (1999) Best management practices for protecting water quality. Proc. Ga. Poultry Conf., University of Georgia, Athens, GA. pp. 89–93.
4. Bandel, V.A. and Griffith, W.K. (1994) Long-term studies revisited — fertilization key to continued productivity. Better Crops with Plant Food **78,** 12–13.
5. Johnson, J.W., Murrell, T.S., and Reetz, H.F., Jr. (1999) Balanced fertility management: a key to nutrient use efficiency. Better Crops with Plant Food **81,** 3–5.
6. Rhoads, F. (1977) Influence of N, P and K on irrigated corn production. University of Florida Research Fact Sheet, UF AREC, Quincy.
7. Anon. (1999) Phosphorus interactions with other nutrients. Better Crops with Plant Food **83,** 11–13.
8. Adams, W.E., White, A.W., McCreery, R.A., and Dawson, R.N. (1967) Coastal bermudagrass forage production and chemical composition as influenced by potassium source, rate and frequency of application. *Agron. J.* **59,** 247–249.
9. Adams, W.E., Elkins, C.B., and Beaty, E.R. (1966) Rooting habits and moisture use of coastal and common bermudagrass. *J. Soil Water Conserv*. **21,** 133–135.
10. Ramsay, B. (2001) Holding carbon for better soils. CTIC Partners **19,** 4–5.
11. Smil, V. (1999) Nitrogen in crop production: an account of global flows. *Global Biogeochem. Cycles* **13,** 647–662.
12. Munson, R.D. and Runge, C.F. (1990) Improving fertilizer and chemical efficiency through "high precision farming". Center for Int. Food and Ag. Policy, U. of Minn., St. Paul. 96 pp.

| | | |
|---|---|---|
| Received: | July | 17, 2001 |
| Revised: | September | 10, 2001 |
| Accepted: | September | 10, 2001 |
| Published: | October | 11, 2001 |
